# Traumatic brain injury complicated by environmental hyperthermia

**DOI:** 10.4103/0974-2700.58660

**Published:** 2010

**Authors:** Erik Hermstad, Bruce Adams

**Affiliations:** Carl R. Darnall Army Medical Center, Fort Hood, Texas, USA; 1 Department of Clinical Investigation, William Beaumont Army Medical Center, El Paso, Texas, USA

**Keywords:** Hyperthermia, resuscitation, traumatic brain injury

## Abstract

Temperature variations after traumatic brain injury are common and devastating. This has been shown most clearly with hypothermia, but the complications associated with hyperthermia in the setting of traumatic brain injury can be just as problematic. We present the case of a soldier with traumatic brain injury exposed to environmental temperatures of 115–120°F with a core temperature of over 108°F. The complications of his conditions are discussed as well as potential treatments for the deadly combination of traumatic brain injury and environmental hyperthermia.

## INTRODUCTION

Temperature variations after traumatic brain injury are common, even within the first several hours following injury. These variations are thought to be due to primary hypothalamus injury and dysfunction as well as increased susceptibility to environmental temperatures, hot or cold, following hypothalamus injury. Admission hypothermia (≤35°C) has been associated with increased odds of death for patients with major trauma and also with isolated severe head injury.[1] However, no case reports or studies could be found concerning admission hyperthermia and outcome for major trauma victims or those with isolated severe head injury. We present the case of a combat trauma victim with near-isolated head injury who presented with an admission core temperature of 42.2° C after being exposed to outdoor temperatures of 46–49°C during prolonged extraction.

## CASE HISTORY

**Figure 1:**
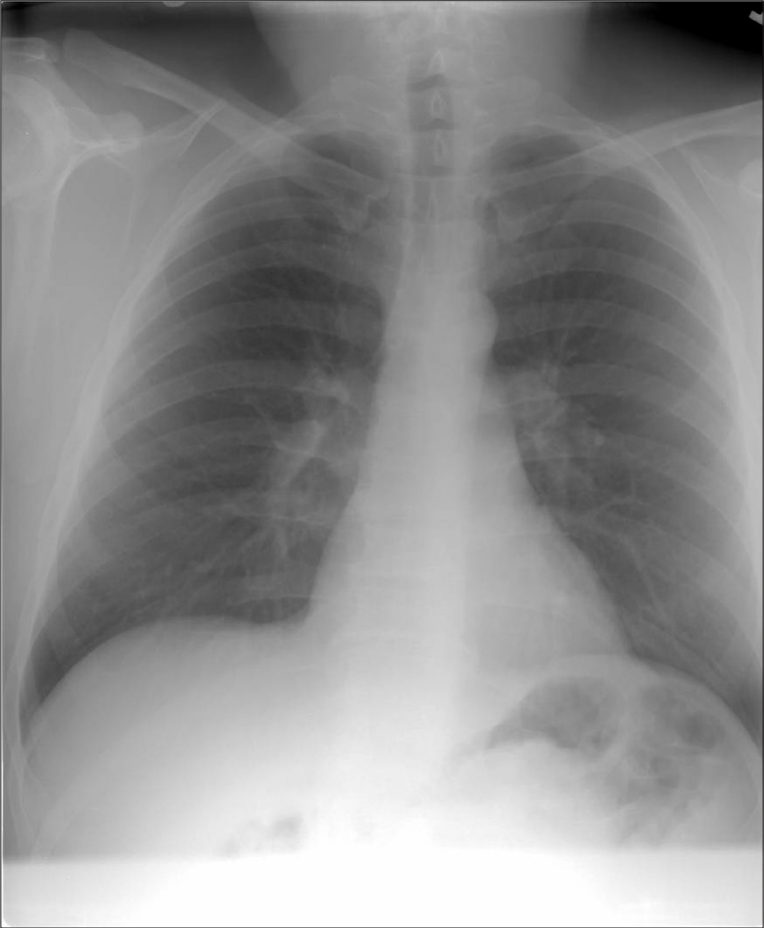
Normal chest X-ray

**Figure 2:**
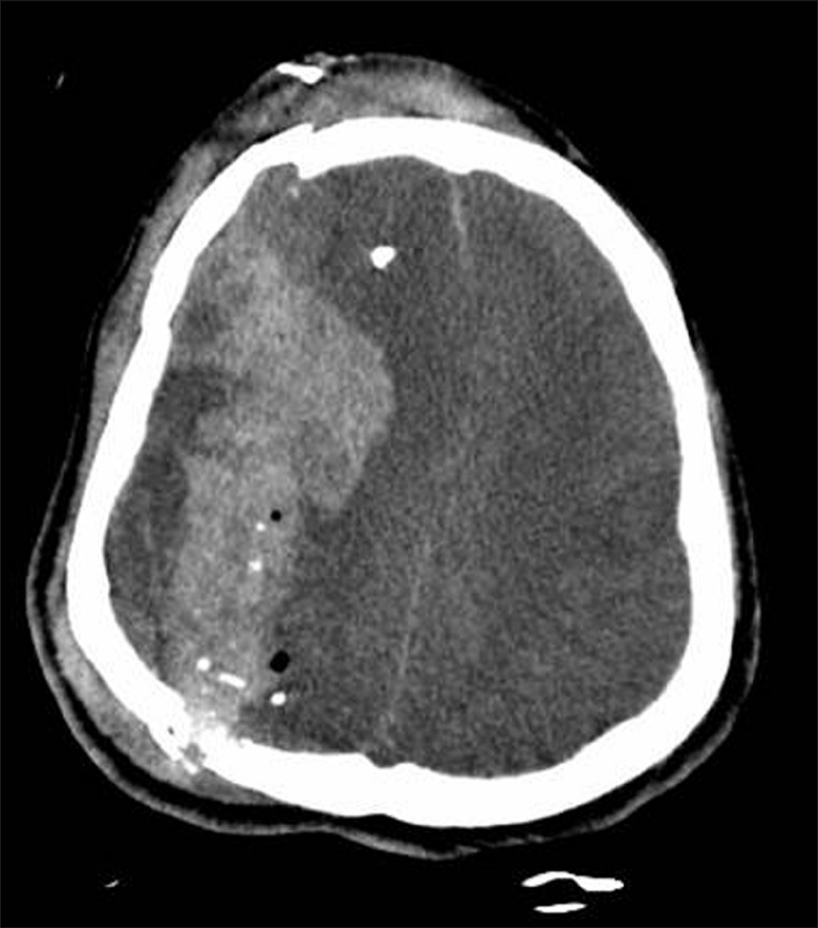
CT image of patients skull fractures and fragmentation with acute intracerebral hemorrhage and midline shift

During the summer in Iraq, an improvised explosive device exploded with a resultant “MASCAL” situation. The temperature outside was 115–120°F. An Iraqi soldier in his twenties was brought to the 228^th^ Combat Support Hospital in Mosul after a somewhat prolonged extraction from a dangerously “hot” combat area. On primary survey, the patient had a patent airway, but with agonal respirations and no response to stimulation; rapid sequence intubation was performed. Bilateral breath sounds were present and carotid pulse was palpable, though faint. The patient's Glasgow coma scale was 3, and pupils were dilated and nonresponsive. Initial vital signs included a rectal temperature of over 108°F, systolic blood pressure of 80, pulse in the 130s, and oxygen saturation of 100% with controlled respirations on the ventilator. Secondary peripheral and central venous catheters were placed and intravenous fluid resuscitation was initiated with lactated ringers. Active cooling was also initiated with ice bags in groin area, axillae, and around the neck. The secondary survey added findings of superficial abrasions, lacerations, and contusions to the right side of the head, face, chest, and right arm. Chest X-ray was performed without significant abnormalities noted [Figure 1]. After 2–3 L of lactated ringers were infused, systolic blood pressure was increased to the low 100–110 range with a decreased pulse to the low 100s. An EKG performed showed sinus tachycardia without other abnormalities. Two units of packed red blood cells were given with continued lactated ringers infusion. The initial arterial blood gas revealed a pH of 7.19, CO_2_ of 65, and a base deficit of –3. Initial hematocrit was 50.6 with a PT of 19.6 and PTT of 36.2. Although the chemistry panel was not available from the limited patient records available, no significant electrolyte abnormalities were noted nor evidence of renal failure. A CT of the head was performed [Figure 2]. With evidence of skull fractures, acute bleeding, and midline shift, the patient's ventilation rate was increased to 20 and 1 g/kg of intravenous mannitol was given. Repeat vital signs before his transfer to the intensive care unit (ICU) included a systolic blood pressure in the 140s, a pulse in the 90s, and a rectal temperature of approximately 100°F. Ice bags were removed from the patient to prevent overcooling. After transfer to the ICU, patient's PT and PTT were noted to have increased to 31 and 293, respectively, and the patient expired 5 h later.

## DISCUSSION

This patient illustrates a deadly case of environmental heatstroke facilitated and potentiated by traumatic brain injury with damage to the hypothalamus. To add insult to injury, the hypercapnia and acidosis evident in this case that commonly develop in heatstroke further impaired thermoregulation and increased coagulopathy; the cumulative result is exhibited in this patient's disastrous outcome.[2,3] Furthermore, the patient's hyperthermia went undetected and untreated during his delayed extraction in a hostile combat environment.

In heatstroke, metabolic acidosis is the predominant acid-base change and is significantly associated with the degree of hyperthermia.[4] As mentioned, the hypercapnia and acidosis that develop in heatstroke serve to impair thermoregulation.[2] This is a setup for a vicious cycle of escalation of temperature and acidosis when such a patient is exposed to prolonged elevated environmental temperature, a common occurrence in the current conflict in Southwest Asia.

Traumatic brain injury is also common on the modern battlefield. Trauma to the skull and brain commonly results in coagulopathy as brain tissue factor is exposed and initiates the coagulation cascade, eventually manifesting as disseminated intravascular coagulation syndrome.[5] Unfortunately for this patient, acidosis also significantly increases coagulopathy.[3] In addition, hyperthermia has been shown in mice to increase hemoglobin extravasation after traumatic brain injury.[6] Combining this data, it might well be said that hyperthermia, acidosis, and coagulopathy are as much the “lethal triad” in traumatic brain injury as hypothermia, acidosis, and coagulopathy are the “lethal triad” in major trauma. For this patient, already with significant acute intracerebral hemorrhage noted on CT, this proved to be true.

In the treatment of such a patient with hyperthermia and traumatic brain injury, active cooling measures are warranted as brain cooling causes attenuation of cerebral oxidative stress, systemic inflammation, activated coagulation, and tissue ischemia and injury. A recent practical review of cooling management in heatstroke found conductive cooling measures, specifically immersion in iced water, to be fast, safe, and effective.[7] Furthermore, texts such as Auerbach's Wilderness Medicine recommend ice-water treatment as the fastest method of cooling.[8] However, for a combat trauma patient who may have multiple penetrating injuries, immersion may be impractical and dangerous. While cooling methods based on evaporation seem to be less efficient than iced water immersion, they are well tolerated, and Bouchama's review suggests that until randomized controlled studies comparing iced-water immersion and evaporative cooling are conducted, the methods should be considered equivalent in treatment of heatstroke. For a patient like this with penetrating trauma, evaporative cooling measures may be more practical. Other cooling methods such as ice packs, cooling blankets, cold-irrigant gastric or rectal lavage, peritoneal dialysis, and cardiopulmonary bypass with a heat exchanger should be considered adjunctive treatments.[9] In this particular case, ice bags were used as a primary cooling method as ice-water immersion was impractical and no appropriately powerful fan was available for facilitating evaporative cooling. Ice bags were removed when the patient's temperature reached approximately 100°F to prevent overcooling. Continuing cooling to the point of therapeutic hypothermia should be avoided in patients with traumatic brain injury as there is no consistent evidence that it improves outcome and its use is associated with pneumonia and coagulopathy.[10]

This patient received typical emergency treatment for traumatic brain injury with adequate oxygenation to keep saturation above 90% and fluid resuscitation to bring systolic blood pressure over 90. With a CT showing midline shift, hyperventilation was started and a bolus of mannitol administered in accordance with recommendations.[11] Alternatively, studies have demonstrated the positive effects of hypertonic saline in the management of head injury and raised intracranial pressure.[12] In addition, smaller volumes of hypertonic saline (3%) have been shown to significantly attenuate heatstroke-induced arterial hypotension, intracranial hypertension, decreased cerebral perfusion, and cerebral ischemia and damage as well as prolong survival time in a rat model of heatstroke.[13] While early studies associated hypertonic saline resuscitation in traumatic brain injury and hypotension with improved outcome, Cooper *et al*. found that hypertonic saline did not improve survival or long-term neurological function compared with conventional resuscitation fluids alone.[14] However, over 90% of these patients had multisystem trauma and it is possible that those with isolated traumatic brain injury respond differently.

Interestingly, 8.4% sodium bicarbonate has recently been reported to be successful in treating raised intracranial pressure in a patient with traumatic brain injury and metabolic acidosis.[15] While the use of sodium bicarbonate can cause an increase in PaCO_2_, the respiratory rate can easily be adjusted in a ventilated patient to compensate for this. In fact, many of these patients, including ours, are already being hyperventilated, making this a nonissue. This may turn out to be an ideal fluid in the management of such patients, but further studies are clearly needed to determine the best resuscitation fluid for a patient with traumatic brain injury, acidosis, and hyperthermia.

This is the first case report of a patient with traumatic brain injury compounded by environmental hyperthermia. Unique to this situation are the seemingly synergistic effects of brain injury and elevated environmental temperatures in facilitating and accelerating core temperature increase, acidosis, and coagulopathy, which can occur during the first day of heatstroke, but is actually more common on the second and third days.[9] Also, this patient illustrates the problems of delayed recognition and treatment of hyperthermia after prolonged extraction and delayed evacuation in a hostile, very hot environment. With consistently high environmental temperatures in the current conflict in Iraq and common traumatic brain injury, this is likely to be more common than has been reported. Unfortunately, combat trauma prehospital data are scarce and reporting is not standardized. Often, a temperature is not obtained until the patient arrives at a medical treatment facility, sometimes hours after the time of injury.

Further investigation is warranted into ideal military protocols for identifying and treating this in the early stages, optimal fluid resuscitation strategy for such patients, and the outcome effects of early prehospital recognition and treatment of hyperthermia with active cooling measures. Clearly, early recognition of temperature aberrations is important, and early intervention with active cooling measures may improve outcome in these patients. For hyperthermic patients such as this with penetrating trauma in remote and hostile environments, evaporative cooling measures should be the mainstay of early therapy and should be initiated before and during evacuation, particularly if evacuation is delayed or prolonged. However, as this case illustrates, adjunctive therapies such as ice bags can be used effectively if other cooling methods are impractical or unavailable. This should help to minimize and prevent the acidosis and coagulopathy that complete this “lethal triad” complicating already devastating traumatic brain injuries on the battlefield. Critical to early recognition and treatment is for medical personnel to be trained to maintain a high index of suspicion for hyperthermia in patients with traumatic brain injury in very hot environments. To this end, future prehospital and field management guidelines for traumatic brain injury might include recommendations for early core temperature measurement and treatment protocols for both hypothermia and hyperthermia.

## CONCLUSION

With damage to the hypothalamus, traumatic brain injury patients are at increased risks for core temperature aberrations. The detrimental effects of hypothermia for these patients have been demonstrated as discussed, but hyperthermia likely confers similar problems for traumatic brain injury patients. We discuss some of these problems through the illustration of a combat trauma patient with traumatic brain injury complicated by environmental hyperthermia. Problems thought to be particularly critical in worsening outcome for these patients include the increased acidosis and worsening coagulopathy accompanying hyperthermia. Active cooling measures must be employed early with a goal of achieving normothermia without overcooling. Key to this is for healthcare providers to maintain a high index of suspicion for core temperature variations in traumatic brain injury patients. Much attention has been given to prevent hypothermia in combat trauma patients, but in the extremely hot environments in which our military personnel are operating, equal attention should be given to prevent hyperthermia, especially in the highly susceptible traumatic brain injury patient, for whom hyperthermia may worsen outcome. While current prehospital and field management recommendations do not include early temperature measurement or abnormal temperature treatment recommendations, this may be something to consider in future editions.[16,17]

The opinions or assertions contained herein are the private views of the authors and are not to be construed as official or as reflecting the views of the Department of the Army or the Department of Defense.
